# Use of Thoracic Ultrasonography to Improve Disease Detection in Experimental BRD Infection

**DOI:** 10.3389/fvets.2021.763972

**Published:** 2021-12-14

**Authors:** Madison M. Porter, Paiton O. McDonald, Jamison R. Slate, Amanda J. Kreuder, Jodi L. McGill

**Affiliations:** Department of Veterinary Microbiology and Preventative Medicine, Iowa State University, Ames, IA, United States

**Keywords:** bovine respiratory disease, thoracic ultrasonography, calves, clinical scoring, pneumonia

## Abstract

Bovine respiratory disease (BRD) is caused by complex interactions between viral and bacterial pathogens, host immune status, and environmental stressors. In both clinical and research settings, current methods for detecting BRD in calves commonly focus on visual indicators such as attitude, nasal discharge, and cough, in addition to vital signs such as rectal temperature and respiration rate. Recently, thoracic ultrasonography (TUS) has become more commonly used in clinical settings, in addition to physical examination to diagnose BRD. To assess the value of performing TUS during experimental BRD infection, 32 calves were challenged with bovine respiratory syncytial virus, to mimic a viral infection, and 30 calves were infected with *Mannheimia haemolytica*, to mimic a bacterial infection. TUS was performed at regular intervals using a standardized method and scoring system in addition to daily clinical scoring. Although overall correlations between clinical scores and TUS scores were generally weak (maximum R^2^ = 0.3212), TUS identified calves with abnormal lung pathology that would have otherwise been misclassified on the basis of clinical scoring alone, both on arrival and throughout the studies. In addition, TUS had an increased correlation with gross lung pathology on necropsy (maximum R^2^ = 0.5903), as compared to clinical scoring (maximum R^2^ = 0.3352). Our results suggest that TUS can provide additional information on calf health at enrollment and throughout a study and may provide an alternative to terminal studies, due to the high correlation with lung pathology at necropsy.

## Introduction

Bovine respiratory disease (BRD) is a leading cause of morbidity and mortality among pre-weaned dairy calves and is a major health concern among feedlot cattle (1, 2). In addition to the cost of treatment, BRD causes economic losses through reduced growth, reduced lifetime milk production, and increased mortality (3, 4). BRD results from a complex interaction between viral and bacterial pathogens, host immune status, and environmental stressors that commonly presents as a primary viral infection followed by a secondary bacterial infection. Early identification is crucial to successful treatment but difficult with current approaches to disease detection. Current detection methods primarily focus on clinical scoring, which relies on indicators such as rectal temperature, difficulty breathing, increased respiration, ear position, and nasal discharge (5). However, these indicators have a low sensitivity and specificity for lesions within the lung (6). In addition, many of these clinical signs such as temperature and depression can be caused by environmental conditions and are nonspecific disease indicators (7). Some clinical scoring methods also fail to identify calves with subclinical pneumonia if they rely on external, visual indicators associated with more obvious clinical disease. Lung auscultation performed by an experienced veterinarian can improve the accuracy of clinical scoring models; however, studies have suggested that this method may lack sensitivity for diagnosing BRD (6), and it is difficult to assign a quantitative score. In the laboratory, research of potential BRD interventions is often performed with controlled infections using clinical scoring as the main method of determining the severity of disease progression. With the current detection methods, many markers of disease severity in these intervention studies can only be observed post-mortem, limiting the ability to analyze disease progression longitudinally.

Thoracic ultrasonography (TUS) has been identified as a rapid, on-farm, validated predictor of lung lesions in pre-weaned dairy calves (8). TUS can identify calves with both clinical and subclinical pneumonia with increased sensitivity and specificity (6, 8). Limited research has been done on the detection of subclinical pneumonia, but calves that suffer from BRD early in life are known to have lower production later in life, such as decreased average daily gain, decreased carcass value, and decreased survival of heifers to first calving (2, 4). These findings suggest that the effects of lung damage persist even after clinical signs return to normal (9). Many studies of TUS and its ability to accurately detect disease within the lungs have taken place in field experiments where the exact infectious agent is unknown. The BRD monitored in these studies is likely complex with coinfections by multiple bacterial or viral agents. Thus, in uncontrolled field settings, there is little opportunity to assess the differences in changes in lung appearance as observed by TUS between bacterial or viral pneumonia and the different ways that these diseases progress within the lung.

The objective of this study was to compare TUS with clinical scoring, gross pathology, and pathogen burden in controlled infection of bovine respiratory syncytial virus (BRSV), to represent the primary viral infection, or *Mannheimia haemolytica*, to represent the secondary bacterial infection, in pre-weaned dairy calves. Our secondary objective was to track the progression of lung lesion development as observed by TUS throughout a controlled infection trial.

## Materials and Methods

### Study Population

This study includes calves from three different trial populations. All animal procedures were conducted in strict accordance with federal and institutional guidelines and were approved by the Iowa State University Institutional Animal Care and Use Committee (IACUC protocols 18-058 and 19-081). Trial 1 consisted of 32 4-week-old, mixed gender Holstein calves that were challenged with aerosolized BRSV. Calves were colostrum-replete with BRSV titers ranging from 32 to 256 (mean 116). Calves were confirmed negative for BVDV before enrollment. Trial 2, also referred to as the Fall 2019 cohort, consisted of 16 4-week-old, mixed gender Holstein calves that were challenged with *Mannheimia haemolytica*. Trial 3, also referred to as the Summer 2020 cohort, consisted of 14 4-week-old, mixed gender Holstein calves that were challenged with *Mannheimia haemolytica*. Antibody titers to *M. haemolytica* ranged from 32 to 128 and did not differ between trials. The animals from all three trials were purchased from the same, single source. The animals were transported approximately 3 h from a farm in Eastern Iowa. Calves were housed individually at the farm but were comingled and house in groups of n = 4 upon arrival at the research facility. Calves were confirmed negative for BVDV before enrollment. Animals from all three trials were housed under AgBSL2 conditions at the Iowa State University Livestock Infectious Disease Isolation Facility. Calves were allowed to acclimate for 4 days before beginning the studies. Animals were fed milk replacer twice daily and had *ad libitum* access to food and water.

### BRSV and *M. haemolytica* Challenge

BRSV inoculum was prepared and administered as previously described (10). Briefly, BRSV strain 375 was prepared from virus stock re-isolated from the lung of an infected animal and passaged on bovine turbinate cells less than four times. The inoculum was determined to be uncontaminated with BVDV by PCR. Calves received the inoculum *via* an aerosolized challenge with ~10^4^ TCID_50_/ml of BRSV strain 375.

*Mannheimia haemolytica* was prepared and administered as previously described (11). Briefly, *M. haemolytica* strains NADC-D153 and NADC-D174 were grown to log phase in Columbia broth for approximately 2.5 h. Growth was then diluted 50-fold for challenge. Calves were challenged intratracheally with 25 ml of *M. haemolytica* in Earle's Balanced Salt Solution (EBSS) at 2 × 10^7^ colony-forming units (CFU)/ml. All preparations were kept on ice before inoculation.

### Clinical Scoring

Calves were scored for clinical illness daily using a modified University of Wisconsin calf health respiratory scoring chart (5). The original scoring chart assigns a number (0–3) to each calf for various clinical signs including fever, ear position, eye discharge, nasal discharge, and cough. Additional scoring categories that were added to the chart include expiration effort (0–3) and lung auscultation (0 = abnormal lungs sound absent, 1 = abnormal lung sounds present) (6). The scores for each day are then totaled, and a total score of ≥5 was used to indicate clinical disease. Clinical scoring was performed by one, blinded individual for the duration of the study. The humane endpoint defined by our IACUC protocol included prolonged increases in body temperature (>40.5° C for more than 48 h), inappetence for more than two feeding periods, and respiratory effort scores of 3 for more than 48 h.

### Thoracic Ultrasonography

**Figure 1:**
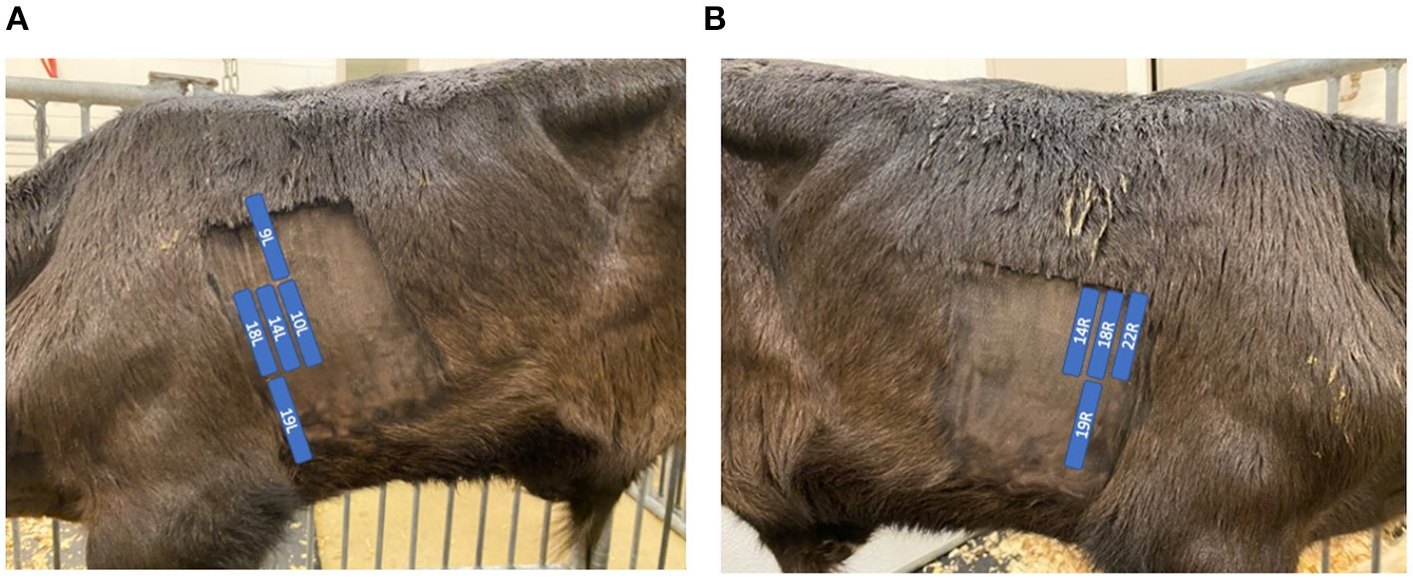
Position of ultrasound probe and description of locations. The labeled blue locations in **(A)** and **(B)** indicate the positioning of the ultrasound probe within each ICS on the left and right sides of the calf, respectively. Location labels are derived from Rademacher et al. (12).

TUS was performed on days 0, 3, 7, 10, and 14 with the BRSV-challenged calves and days 0, 1, 2, 3, and 4 with the *M. haemolytica*–challenged calves. TUS was performed with an IBEX® EVO® (E.I. Medical Imaging, Loveland, CO) using the L7HD linear transducer probe (5–9 MHz) set to a depth of 8.7 cm for all scans with 70% isopropyl alcohol applied to the areas of interest. The thoraxes of all calves were clipped within 1 week before the start of challenge to improve image quality. Nine locations on the calf were identified for ultrasonography with five locations on the left side of the calf and four locations on the right side of the calf. Ultrasound locations were based on previously described locations of interest (12) and are shown in Figures 1A,B. Locations were observed in the same order each time beginning with the most cranial and ventral location on the left side and moving caudally and dorsally on the left side and then repeated on the right side. The first ultrasound location on the left side of the calf was identified by placing the probe so that the bottom of the probe was even with the point of the elbow and in the intercostal space (ICS) immediately caudal to the shoulder muscle. The remaining four locations on the left side of the calf were placed, as described in Table 1. On the right side of the calf, the first ultrasound location was found by again placing the probe in the ICS that was immediately caudal to the shoulder muscle with the first location on the right side even with the point of the shoulder. The remaining three locations on the right side of the calf were placed, as described in Table 1. At each location, a four second clip was recorded and stored for later image review. Images were captured by study personnel without previous ultrasound experience (MP and PM) but who were trained on ultrasound image collection by an experienced ultrasonographer [AK; diplomate, American College of Veterinary Internal Medicine (Large Animal)] at the beginning of trial 1.

**Table 1:** Anatomical landmarks associated with each ultrasound location.

**Location name**	**Anatomical landmarks**
19L	ICS immediately caudal to shoulder muscle, bottom of probe even with point of elbow
18L	One probe length dorsal to 19L, middle of probe even with point of shoulder
14L	One ICS caudal from 18L, middle of probe even with point of shoulder
10L	One ICS caudal from 14L, middle of probe even with point of shoulder
9L	One probe length dorsal to 10L, bottom of probe even with point of shoulder
22R	ICS immediately caudal to shoulder muscle, middle of probe even with point of shoulder
18R	One ICS caudal from 22R, middle of probe even with point of shoulder
19R	One probe length ventral to 18R, bottom of probe even with point of elbow
14R	One ICS caudal to 18R, middle of probe even with point of shoulder

*L in the location name denotes the location was on the left side of the calf and R in the location name denotes a location on the right side of the calf. Ultrasounds were always taken in the order listed*.

### Image Review

Images were scored by a reviewer without previous ultrasound experience (MP) who was trained on ultrasound interpretation by an experienced ultrasonographer (AK). Initial training consisted of review of images concurrently for identification of lung consolidation, pleural defects including comet tail artifacts and B-lines, pleural irregularity, pulmonary abscessation, and pleural fluid presence. A subset of images was then scored independently by the trained ultrasonographer and the trainee, and results were compared and confirmed to be consistent between reviewers. All ultrasound scoring utilized in this study was thereafter performed by the trainee; the final review of any substantially discordant results between necropsy scoring and TUS was also performed on as needed basis by the experienced ultrasonographer. Ultrasound clips for each location were scored for presence of pleural defects and depth of consolidation. Pleural defects were assigned a score of 0–3, 0 = no comet tails/B-lines observed; 1 = one to two comet tails/B-lines observed; 2 = three to five comet tails/B-lines observed; 3 = six and above comet tails/B-lines observed. Depth of consolidation was also assigned a score of 0–3 based on the maximum depth of consolidation seen at each location: 0 = no consolidation, 1 = <2 cm of consolidation, 2 = >2 cm but <4 cm of consolidation, and 3 = >4 cm of consolidation. Once all nine locations for a day were scored, the scores for presence of pleural defects were combined for a final pleural defect score and the maximum consolidation depth seen in any of the nine locations on each day was assigned as the final depth of consolidation score. Calves were assigned a final ultrasound score for the day on a scale of 0–4: 0 = no consolidation present, final pleural defects score of <5; 1 = no consolidation present, final pleural defects score >5; 2 = final depth of consolidation score of 1; 3 = final depth of consolidation score of 2; 4 = final depth of consolidation score of 3.

### Necropsy

BRSV-challenged calves were humanely euthanized on day 14 after infection or as clinical symptoms deemed necessary. *M. haemolytica*–challenged calves were euthanized on day 4 after infection or as clinical symptoms deemed necessary. All were euthanized by barbiturate overdose using sodium pentobarbital administered at 100 mg/kg. On necropsy, the lungs were removed, and 11 lobes (left cranial, right cranial, middle, upper right caudal, middle right caudal, lower right caudal, lower left caudal, middle left caudal, upper left caudal, accessory, and left cranial caudal lobes), as shown in Supplementary Figure 1, were assessed for the presence of lesioned lung on a percent basis. Lung tissue was assessed for any visual changes to the surface of the lung and palpated for any changes to lung texture. Necropsy data were not included from BRSV-challenged calves that were euthanized before day 14. To identify the lung lobes that were captured on ultrasound, the calf was placed on its side, the ultrasound locations were identified, as shown in Supplementary Figures 2A,B, and Trypan blue dye was injected into the lung tissue. Once the lungs were removed from the calf, the blue injection sites were identified and marked, as shown in Supplementary Figures 2C,D. Five lobes of interest were identified: left cranial, left cranial caudal, upper left caudal, right cranial, and middle.

### Real-Time PCR and Quantitative Culture

Viral load was determined in lung tissue and nasal swab samples by quantification of BRSV NS2 copy number, as previously described (13).

Briefly, randomly selected samples, ~0.5 g each, lesioned and non-lesioned lung tissues were collected from two separate lung lobes each calf and stored in RNAlater (ThermoFisher). RNA was then isolated and pooled from the separate locations (lesioned samples pooled together, non-lesioned lung tissues pooled together) using Trizol Reagent (Life Technologies) and then cleaned up using a Qiagen RNeasy isolation column, as described (14). Nasal swabs samples were collected from the upper nasal cavity of BRSV infected calves on days 0, 3, 7, 10, and 14 after infection. Viral RNA was isolated from nasal swab samples using the MagMax Viral Isolation Kit (Applied Biosystems, Life Technologies). cDNA synthesis and quantitative rtPCR reactions were carried out with the TaqMan RNA-to-CT 1-step-kit (Applied Biosystems) according to the instructions of the manufacturer. Primers and probes for the BRSV NS2 gene and the bovine RPS9 gene have been published (13). Reactions were performed using a ThermoFisher QuantStudio 3 Real-Time PCR machine under previously described cycling conditions (10). Standard curves for NS2 and RPS9 genes were run in parallel with test samples, and all were run in duplicate. Viral NS2 copy numbers were calculated using standard curves and normalized to RPS9 to account for differences in input materials.

Quantitative culture for *M. haemolytica* was performed, as previously described (11). Briefly, lung tissue samples were ground in EBSS to produce a homogenized suspension that was then diluted 10-fold in EBSS. The dilutions were then spread on blood agar base plates containing 5% defibrinated bovine blood and incubated overnight at 37°C. Colonies with typical *M. haemolytica* morphology were enumerated, and representative colonies were selected for plate agglutination. Cotton-tipped applicators were rolled on half of a fresh blood agar plate then a sterile loop was used to semi-quantitatively streak for isolation on the remaining two quarters.

### Statistical Analysis

Statistical analysis was performed using Prism v9.0.1 (GraphPad Software, Inc.). Data were analyzed using linear regression and R values were calculated using the Pearson method.

## Results

### Infection Models

**Figure 2:**
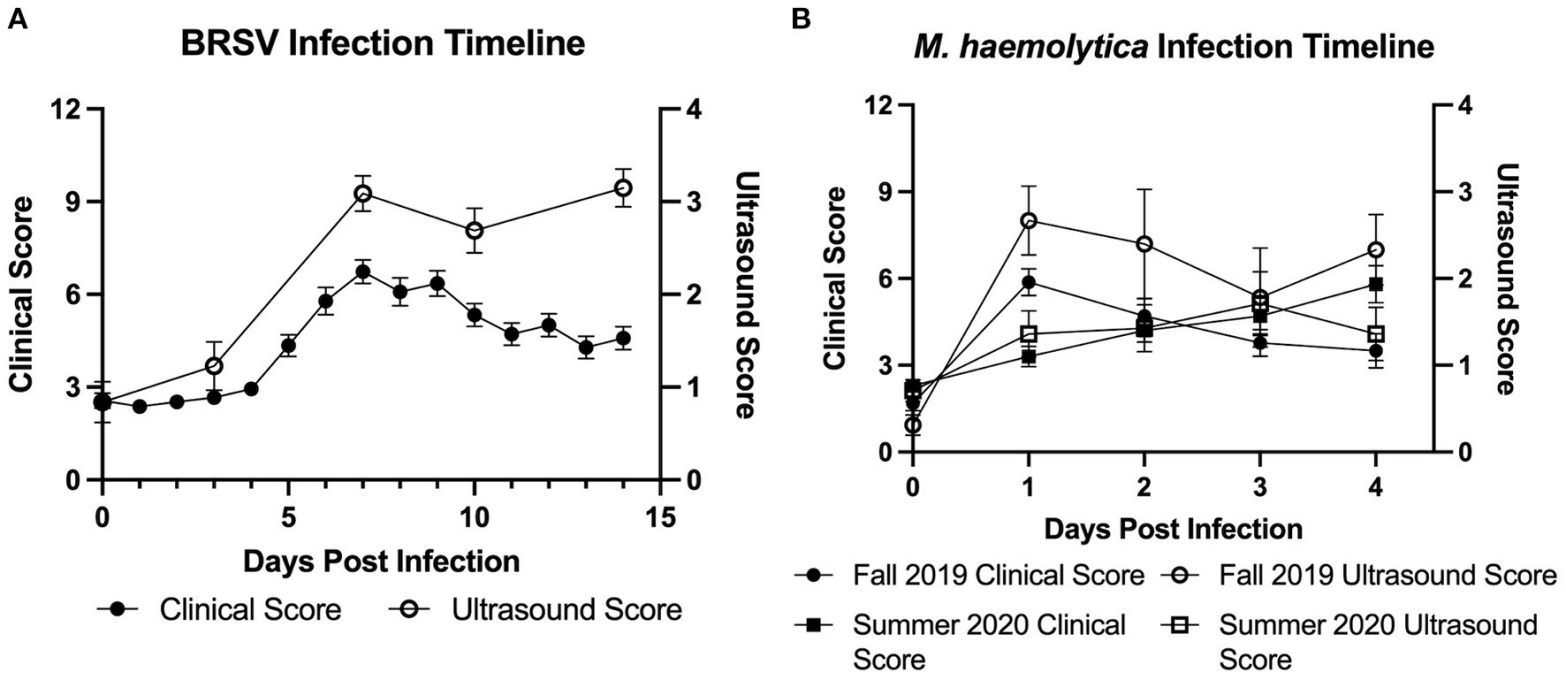
Timeline of experimental BRSV and *M. haemolytica* infection. Calves were challenged with either BRSV **(A)** or *M. haemolytica* **(B)**. Unless clinical signs necessitated earlier euthanasia, calves challenged with BRSV were euthanized on day 14 after infection, whereas calves challenged with *M. haemolytica* were euthanized on day 4 after infection. All calves were assigned a clinical score each day by a trained observer based on fever, eye and nose discharge, severity of lung sounds, and ear position. All calves were assigned an ultrasound score on selected days (days 0, 3, 7, 10, and 14 in BRSV-challenged calves; all days in *M. haemolytica*–challenged calves) by a trained reviewer based on presence and severity of consolidated lung tissue and pleural defects such as B-lines and comet tail artifacts. Data represent mean ± SEM.

In the first trial, 32 4-week-old, mixed gender Holstein calves were infected with BRSV strain 375 *via* aerosol inoculation. Calves were monitored daily and assigned a clinical disease score on the basis of an adapted University of Wisconsin calf health scoring model. TUS images were collected on days 0, 3, 7, 10, and 14 after infection. Because of elevated clinical disease scores, two calves were euthanized on day 6, and four calves were euthanized on day 7. The remaining 26 calves were euthanized on day 14 after infection. As shown in Figure 2A, both clinical disease and TUS scores peaked on day 7 after infection. Clinical scores gradually decreased through day 14; however, ultrasound scores remained elevated for the remainder of the study.

In the second and third trials, a total of 30 4-week-old, mixed gender Holstein calves were infected *via* intratracheal inoculation with 10^7^ CFU *M. haemolytica* strain NADC-D153. Calves were monitored and assigned a clinical disease score daily with TUS images collected daily on days 0–4 after infection. In trial 2, six calves were euthanized on day 1, one calf was euthanized on day 2, and the remaining nine calves were euthanized on day 4 after infection. In trial 3, all 14 calves were euthanized on day 4 after infection. The Fall 2019 cohort of *M. haemolytica–*infected calves had peak clinical and ultrasound score on day 1 after infection, and the Summer 2020 cohort had clinical scores and TUS scores that peaked on days 3 and 4, respectively, as shown in Figure 2B.

### Disease Progression

All calves had TUS performed before inoculation with either BRSV or *M. haemolytica*. In trial 1, seven of the 32 BRSV-infected calves had a TUS score ≥2 on arrival indicating the presence of previous clinical disease or current subclinical pneumonia. In trials 2 and 3, none of the 30 *M. haemolytica*–infected calves had a TUS ≥ 2 on arrival.

**Figure 3:**
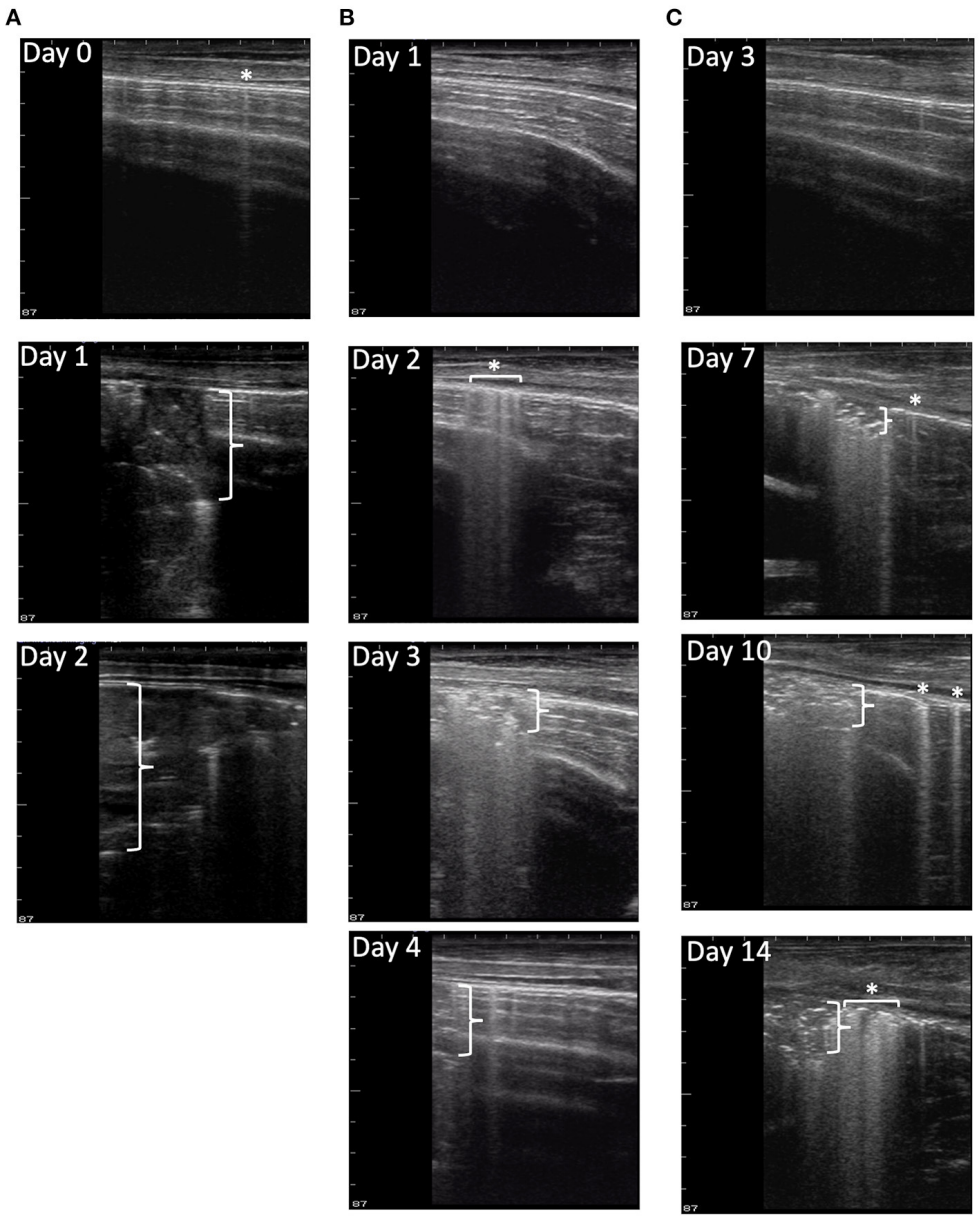
Progression of disease in ultrasound images. Ultrasound images were collected from nine locations each day for each calf in the study. **(A)** depicts ultrasound image progression from location 14R on days 0, 1, and 2 of a *M. haemolytica*–infected calf from the Fall 2019 cohort. The final TUS scores of the calf were 0, 4, and 4, and its clinical scores were 2, 7, and 9, respectively. The calf was euthanized on day 2 after infection because of the severity of disease progression. **(B)** depicts the ultrasound image progression from location 18L on days 1, 2, 3, and 4 of a *M. haemolytica*–infected calf from the Summer 2020 cohort. The final TUS scores of the calf were 1, 1, 4, and 4, and its clinical scores were 4, 4, 7, and 2, respectively. The calf was euthanized on day 4 after infection. **(C)** depicts the ultrasound image progression from a location 18L on days 3, 7, 10, and 14 of a representative BRSV-infected calf. The final TUS scores of calf were 0, 3, 3, and 3, and its clinical scores were 1, 9, 6, and 2, respectively. The calf was euthanized on day 14 after infection. Asterisks with or without horizontal brackets indicate pleural defects and vertical brackets indicate maximum depth of consolidation.

After experimental infection, consistent trends, unique to each disease process, were observed in the development of pleural defects and consolidation that were detected by TUS as shown in Figure 3. In *M. haemolytica–*infected calves, a majority of calves (12/16) in the Fall 2019 cohort (Figure 3A) developed lesions consistent with consolidation (TUS score ≥ 2) on TUS within 24 h of infection. After the peak observed on day 1 after infection, the average ultrasound score steadily decreased until the final observation on day 4 after infection. Notably, however, six of the most severe calves were euthanized 24 h after infection, and an additional two were euthanized 48 h after infection, including the calf depicted in Figure 3A, which likely contributed to the decrease in ultrasound score as the study progressed. In the Summer 2020 cohort (Figure 3B), one third of the calves (5/15) developed lesions consistent with consolidation on TUS within 24 h of infection and one additional calf developed lesions consistent with consolidation by day 4 of the study. No calves were euthanized before necropsy on day 4 after infection. In general, the consolidation lesions observed on ultrasound in the *M. haemolytica*–infected calves had well-defined borders; this was consistent with the appearance of localized, firm lesions observed in the lung tissue on necropsy.

In the calves that had no prior consolidation, lesions consistent with lung consolidation (TUS score ≥ 2) were commonly first identified *via* TUS on day 7 after BRSV infection and remained detectable until necropsy (Figure 3C). The calves with positive TUS scores before infection were no more likely to develop positive clinical scores or elevated TUS scores; however, these elevated scores were detected sooner. In addition, pleural defect scores peaked on day 10 after infection and were not always associated with the presence of consolidation. Lesions consistent with consolidation observed on ultrasound in BRSV infection had less defined borders than those of the *M. haemolytica*–infected calves; this was also consistent with the appearance of more diffuse, regionalized lesions within the lung tissue on necropsy.

### Clinical Score and Ultrasound Score

**Figure 4:**
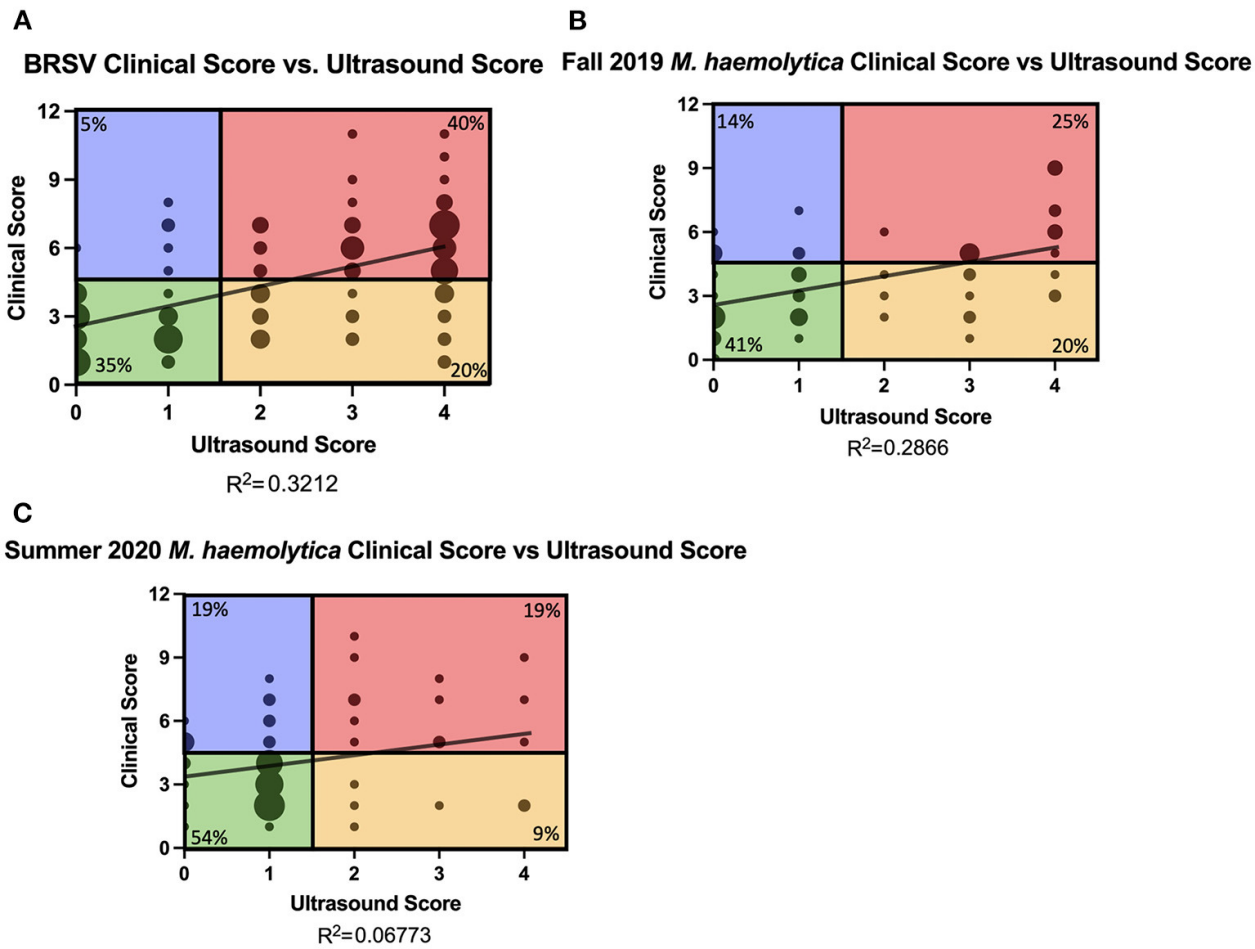
Relationship of clinical score and TUS score and classification of calves. Daily clinical scores were compared to daily ultrasound scores from BRSV-infected calves **(A)** and *M. haemolytica*–infected calves **(B,C)**. The graphs include all data points from days 0, 3, 7, 10, and 14 in BRSV-infected calves and from days 0 to 4 in *M. haemolytica*–infected calves. The size of the point represents the number of calves represented in each location: **(A)** the maximum dot size represents 10 calves; **(B)** the maximum size dot represents seven calves; **(C)** the maximum sized dot represents 12 calves. The plots were divided into four quadrants according to positive clinical score (≥5) and positive ultrasound score (≥2). Green quadrant represents calves that are negative for clinical score and ultrasound score. Blue quadrant represents calves with positive clinical scores but negative ultrasound scores. Red quadrant represents calves with elevated clinical and ultrasound scores (clinical). Yellow quadrant represents calves who had non-elevated clinical scores but had positive ultrasound scores (subclinical). The percentage in each quadrant represents the frequency of points from that study represented in each quadrant.

Diagnosing BRD through clinical disease scoring relies primarily on visual cues and an elevated body temperature. A number of field studies have demonstrated the poor sensitivity of clinical disease scoring systems, which fail to detect a significant population of calves with subclinical pneumonia (6). Few studies have compared the relationship between clinical disease scoring progression with TUS scoring progression in controlled infection settings. Therefore, we compared clinical disease score and TUS score from all calves from all days of the study. In trial 1, 131 clinical disease score and TUS score pairs from all days that TUS images were collected were compared (Figure 4A). In trials 2 and 3, 59 and 69 clinical disease score and TUS score pairs from all days of the study were compared (Figures 4B,C). In the BRSV infection model, there was a weak to mild positive correlation between clinical disease score and TUS score (R^2^ = 0.3212). Given the differences in disease progression, the *M. haemolytica*–infected calves from trials 2 and 3 were treated as two separate cohorts. The correlation between clinical disease score and TUS score was lower after *M. haemolytica* infection, regardless of cohort (R^2^ = 0.2866 in the Fall 2019 cohort and R^2^ = 0.0677 in the Summer 2020 cohort).

To further explore the relationship between clinical disease score and TUS score, the scatterplots were then divided into four quadrants on the basis of a positive clinical score (CS≥5) and the presence of consolidation (TUS score≥ 2). The bottom left quadrant represents calves with negative clinical scores and no consolidation present; these calves make up 41% (107 of 259) of all the data points collected. The top left quadrant represents calves with a positive clinical score but with no consolidation present; these calves make up 10% (27 of 259) of the data points collected. The top right quadrant represents calves with a positive clinical score and consolidation present; these calves have clinical pneumonia and make up 31% (81 of 259) of the data points collected. The bottom right quadrant represents calves with a negative clinical score but consolidation present; these calves have subclinical pneumonia that is undetectable by other methods of disease progression monitoring and make up 17% (44 of 259) of the data points collected.

**Figure 5:**
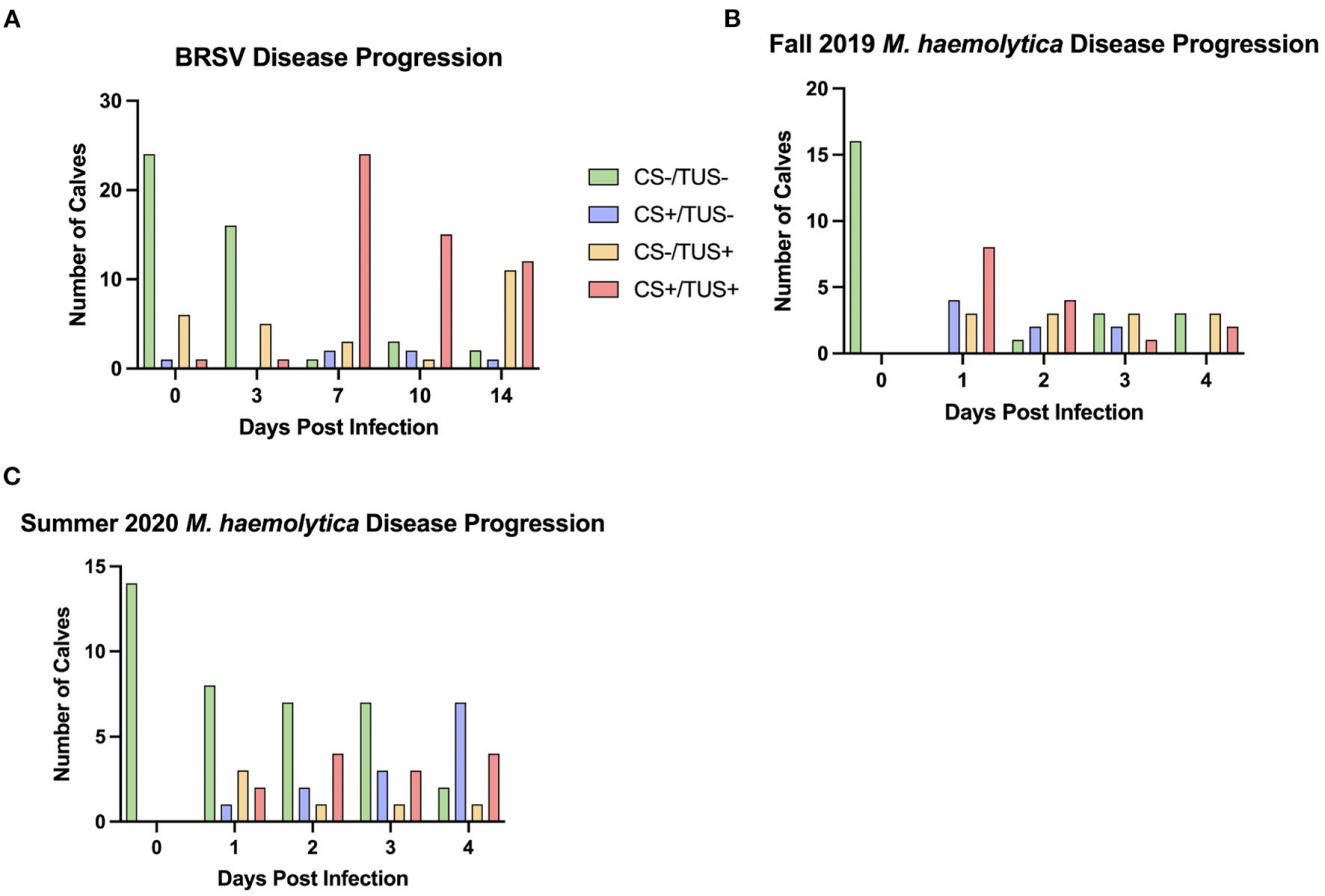
The distribution of calves represented in each of the four quadrants over the course of BRSV **(A)** and *M. haemolytica* **(B,C)** infection. For the BRSV-infected calves **(A)**, TUS was performed on days 0, 3, 7, 10, and 14 after infection. For the *M. haemolytica*–infected calves **(B,C)**, TUS was performed on days 0, 1, 2, 3, and 4 after infection.

Of the 32 BRSV-infected calves, 19 were classified as having subclinical pneumonia on at least one day that TUS was performed. The highest incidence of subclinical pneumonia, 11 of the 26 remaining calves, occurred on day 14 after infection (Figure 5A). On day 0 of the study, 24 calves had negative clinical and TUS scores. By day 7 after infection, 24 calves were in the clinical pneumonia classification with positive clinical and TUS scores. Six of these calves were euthanized before day 10 because of severe clinical disease symptoms, so on day 10 after infection, 15 calves remained in the clinical pneumonia classification. By day 14, the number of clinical pneumonia calves was reduced to 12, and there were 11 calves classified with subclinical pneumonia (Table 2). On average, the calves had negative clinical disease and TUS scores on days 0 and 3 after infection and then entered the clinical pneumonia classification on day 7 after infection. The calves that survived to day 10 generally continued to present with clinical pneumonia scores and then moved into the subclinical pneumonia classification by day 14 after infection.

**Table 2:** TUS and clinical score progression of calves across BRSV and *M. haemolytica* infection.

**BRSV cohort**		Day 0	Day 3	Day 7	Day 10	Day 14
	CS(–)/TUS(–)^a^	24	16	1	3	2
	CS(+)/TUS(–)	1	0	2	2	1
	CS(–)/TUS(+)	6	5	3	1	11
	CS(+)/TUS(+)	1	1	24	15	12
***M. haemolytica*** **Fall 2019 cohort**		Day 0	Day 1	Day 2	Day 3	Day 4
	CS(–)/TUS(–)	16	0	1	3	3
	CS(+)/TUS(–)	0	4	2	2	0
	CS(–)/TUS(+)	0	3	3	3	3
	CS(+)/TUS(+)	0	8	4	1	2
***M. haemolytica*** **Summer 2020 cohort**		Day 0	Day 1	Day 2	Day 3	Day 4
	CS(–)/TUS(–)	14	8	7	7	2
	CS(+)/TUS(–)	0	1	2	3	7
	CS(–)/TUS(+)	0	3	1	1	1
	CS(+)/TUS(+)	0	2	4	3	4

a *CS(–)/TUS(+) is also referred to as subclinical pneumonia. CS(+)/TUS(+) is also referred to as clinical pneumonia*.

In the two trials with *M. haemolytica*–infected calves, seven of the 16 calves in the Fall 2019 cohort and four of the 14 calves in the Summer 2020 cohort were classified as having subclinical pneumonia on at least one day that TUS was performed (Figures 5B,C). In these two trials, the highest incidence of subclinical pneumonia was on day 1 after infection, with six of the 30 calves in this classification. In trial 2, all 16 calves began the study with negative clinical and TUS scores. On day 1 after infection, half the calves were classified as having clinical pneumonia and four calves had positive clinical scores with negative TUS scores. Six of these animals were euthanized because of severe clinical disease symptoms. By day 2 after infection, calves were mostly distributed in the subclinical and clinical pneumonia classifications. An additional calf was euthanized because of severe symptoms. On days 3 and 4 after infection, approximately half the calves had negative clinical and TUS scores, and the remainder was classified with subclinical pneumonia. In trial 3, all 14 calves entered the study with negative clinical and TUS scores. On day 1 after infection, eight calves remained in this classification and the remaining six were distributed among the other three classifications. On day 2 after infection, seven calves were still negative in both scores, but four calves had entered the clinical pneumonia classification, and similar numbers were seen on day 3 after infection. By day 4 after infection, only two calves remained negative for both clinical and TUS scores, seven calves had positive clinical scores and negative TUS scores, and four calves were in the clinical pneumonia classification. By using TUS, calves that lacked clinical symptoms but still developed lung consolidation after experimental infection were frequently identified.

### Pathogen Burden and Ultrasound Score

**Figure 6:**
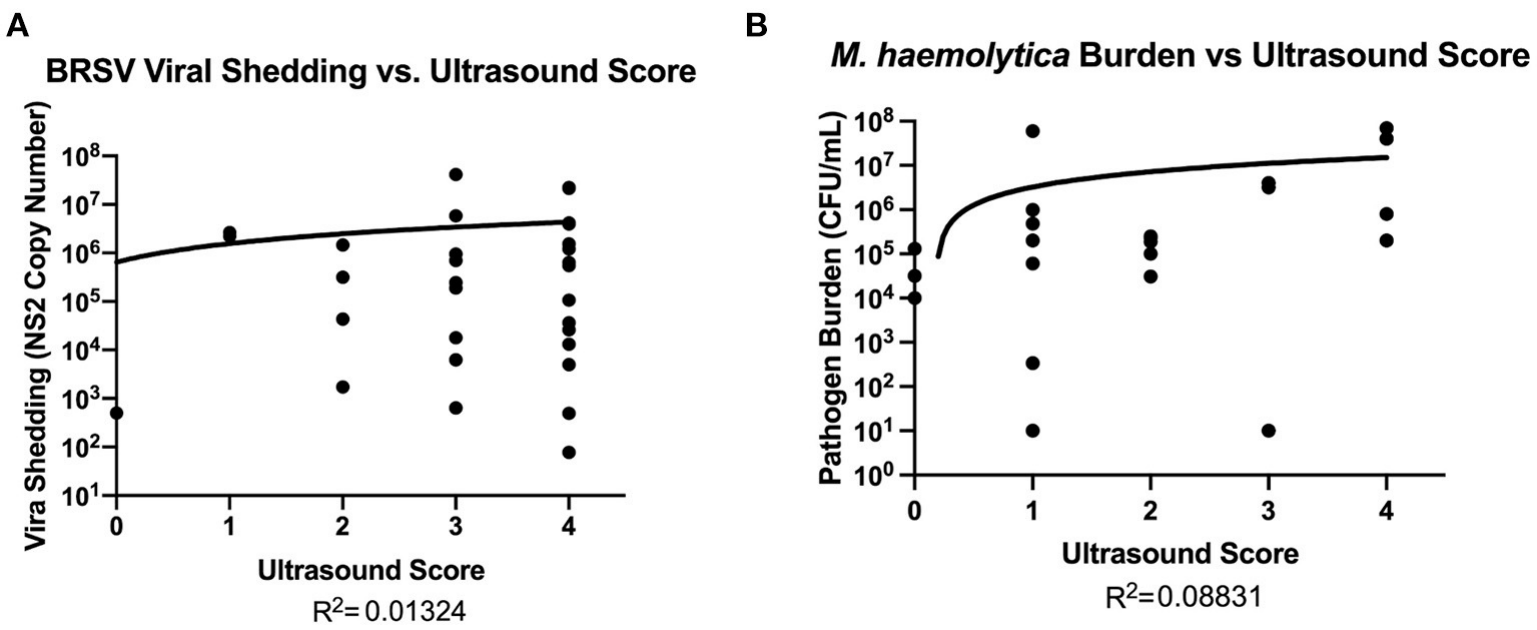
Relationship between pathogen burden and TUS score. For BRSV–infected calves **(A)**, qRT-PCR was performed on nasal swabs for the BRSV NS2 gene to quantify the amount of virus being shed in nasal secretions on day 7. For *M. haemolytica*–infected calves **(B)**, quantitative culture was performed using samples of lung tissue to estimate bacterial lung burden.

Although it is known that a portion of the lung damage arising during BRD is a result of immunopathology, there is little information available regarding the relationship between the development and progression of lung lesions and pathogen load. Therefore, nasal swabs samples were collected from BRSV-infected calves for quantification of viral shedding in Trial 1, and representative lesioned and non-lesioned lung tissues were collected for evaluation of pathogen burden in Trials 2 and 3. In Figure 6A, quantitative Real-Time (qRT)-PCR was performed for the BRSV NS2 gene on nasal swabs collected from the BRSV-infected animals on day 7 after infection, the peak of virus shedding in this model (15). Virus quantity, described as copy numbers of the NS2 gene, was then plotted against the ultrasound scores. There was no association between ultrasound scores and peak virus shedding on day 7 after infection (R^2^ = 0.01324). Samples of lesioned and non-lesioned lung tissue were also collected from the BRSV-infected calves on day 14 after infection. As seen in Supplementary Figure 3, few BRSV NS2 gene copies were detected at this timepoint, and there was no association between TUS score and lung viral load at day 14 after infection (R^2^ = 0.03588 in non-lesioned lung tissue and R^2^ = 0.02543 in lesioned lung tissue). Lung tissue samples were collected from *M. haemolytica*–infected calves at necropsy, and lung bacterial loads were determined by quantitative culture. As shown in Figure 6B, lung bacterial loads ranged from undetectable to as high as 7 × 10^7^ CFU/g of lung tissue. The lung bacterial loads depicted in Figure 6B include calves that were euthanized at humane endpoint and thus include samples that were collected on days 1 (six calves), 2 (one calf), and 4 (23 calves) after infection. There was no association between *M. haemolytica* lung burden at necropsy and ultrasound score (R^2^ = 0.08831). Thus, there is no discernible relationship between pathogen load and TUS score at the peak of virus shedding (day 7 after infection) for BRSV-infected calves or at the day of necropsy for *M. haemolytica*–infected calves.

### Pathology and Clinical Score

**Figure 7:**
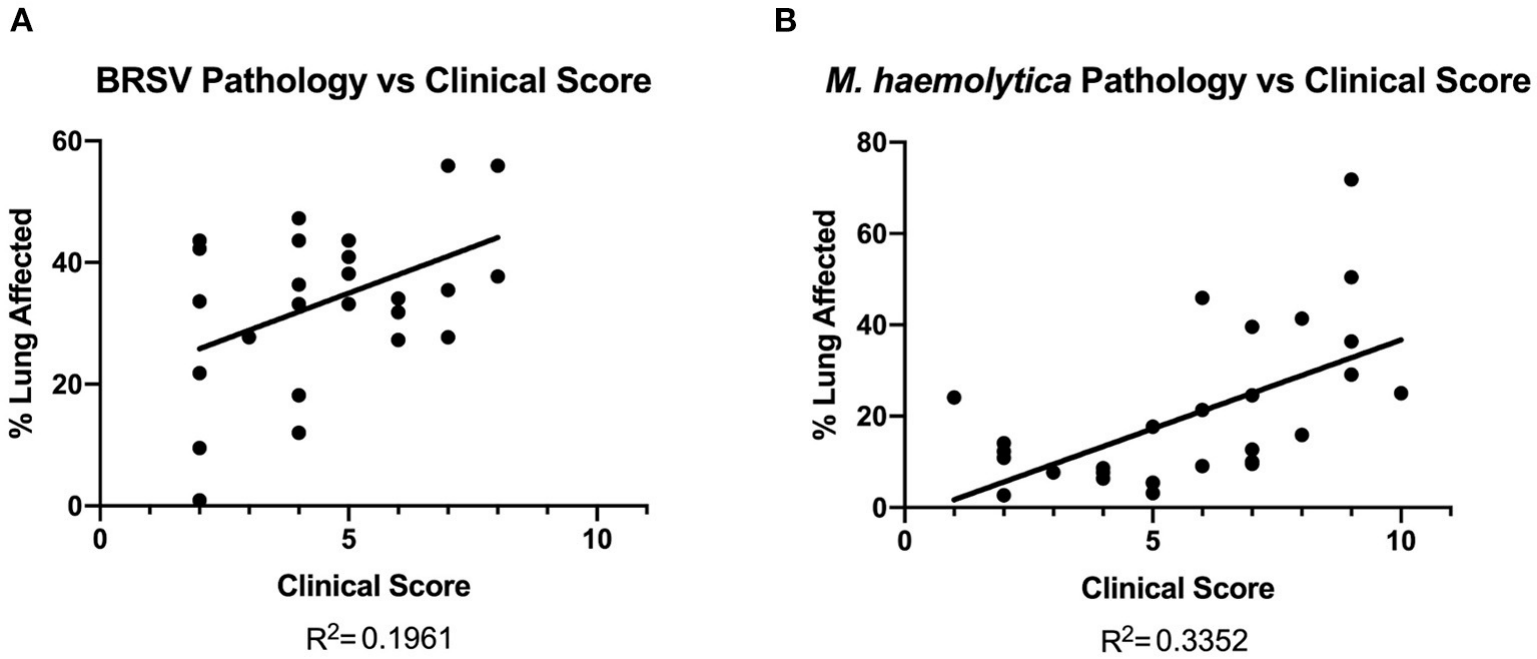
Gross lung pathology on necropsy plotted against clinical score on day of necropsy. In both BRSV-infected **(A)** and *M. haemolytica*–infected calves **(B)**, the lungs were divided into 11 lobes and scored based on percentage of lung affected by gross lesions. There was a weak correlation between clinical score and gross lung pathology in both models.

Lung tissue damage can have long-term effects on calf health and performance. Because clinical disease scoring is a widely used disease detection model, its correlation to gross lung pathology can provide a useful basis for determining whether TUS is more or less accurate in detecting the presence of lesions in the lung tissue. All calves that had necropsy data collected from all three studies showed visible lung lesions on necropsy. In Figure 7, the area of pneumonic lung, expressed as a percent, was compared to clinical disease score on the day of necropsy. There was a weak to mild positive correlation among BRSV-infected calves (R^2^ = 0.1961). In the *M. haemolytica*–infected calves, the relationship between clinical disease score and gross lung pathology score was stronger (R^2^ = 0.3352) but still failed to show a strong relation between the two parameters. This result is not unexpected and underlines the point that clinical disease score is not a strong predictor of the disease severity in the lung. Therefore, for researchers who want to determine the efficacy of a BRD intervention, clinical disease scoring on its own only provides limited information related to disease progression and severity.

### Pathology and Ultrasound Score

**Figure 8:**
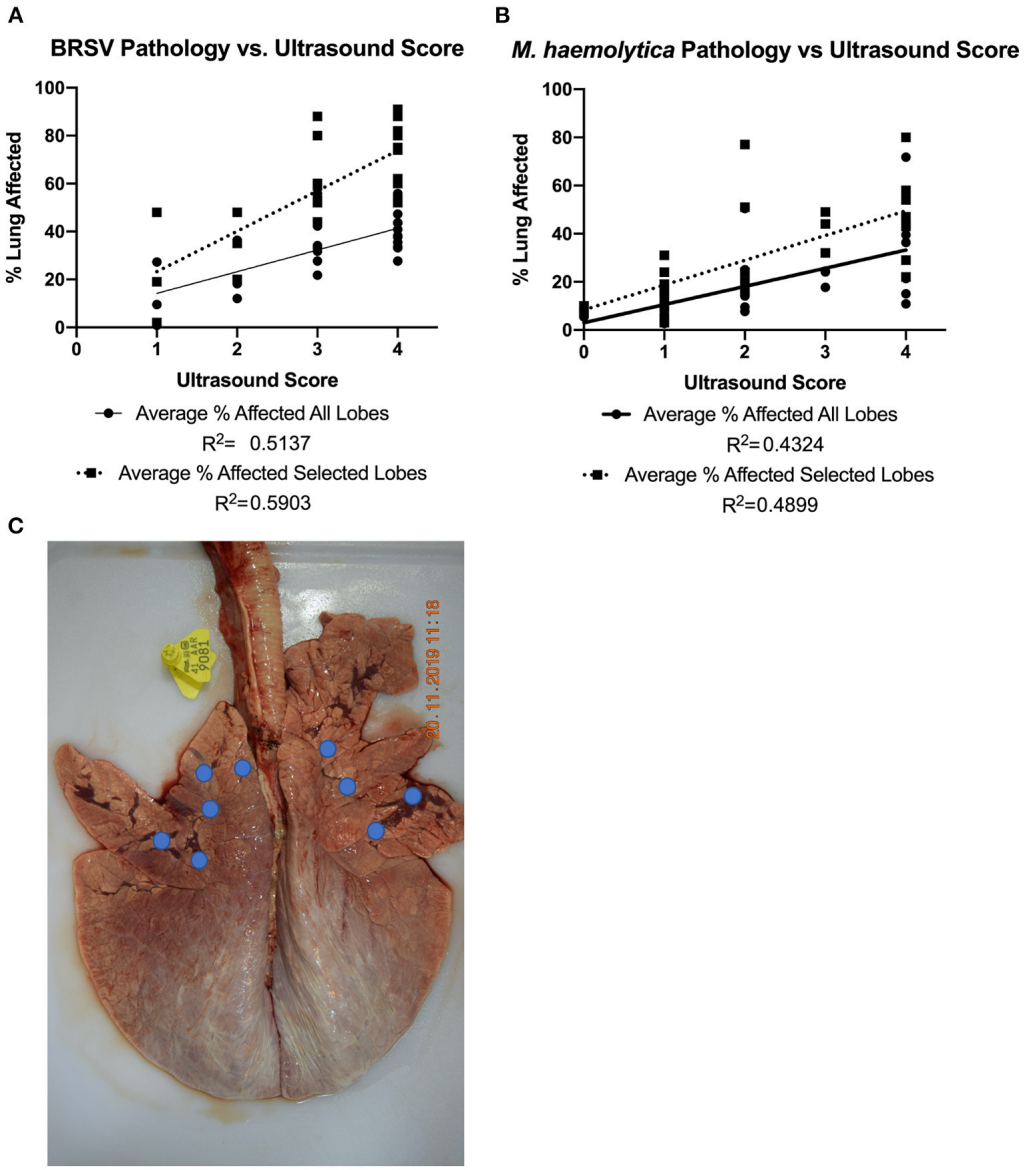
Gross lung pathology on necropsy plotted against ultrasound score on necropsy. In both BRSV-infected **(A)** and *M. haemolytica*–infected calves **(B)**, the lungs were divided into 11 lobes and scored based on percentage of lung affected by gross lesions. The average percent affected across all lobes was plotted in addition to an average across the five lobes imaged *via* ultrasound. The approximate ultrasound locations for both the left and right sides of the lung are shown **(C)**. Because the entire lung field was examined *via* TUS, the average of the lobes that were imaged was plotted separately. In both disease models, there was a correlation between gross lung pathology on necropsy and ultrasound score and the correlation increased when only the lobes with the TUS locations were used.

To further explore the accuracy and sensitivity of TUS scores for predicting gross lung damage, the correlation between ultrasound score on necropsy and gross lung pathology score was determined in Figures 8A,B. The nine ultrasound locations that were evaluated were focused in the cranial and ventral regions of the lung field because consolidation is more likely to develop in these lobes. One caveat of the ultrasound system is the restricted depth of penetration if normal lung tissue is present. This limits the ability of the ultrasound to detect lesions that are deep to normal aerated lung and certain regions of the lung. Therefore, in addition to comparing the total percentage of lung affected, the percentage of pneumonic lung within the five cranial and ventral lobes of interest that were directly examined using ultrasound (as indicated by the location highlights in Figure 8C) was also calculated and compared to the respective TUS score. A moderate correlation between the gross lung pathology score encompassing the entire lung and TUS score on necropsy was observed for calves infected with BRSV (R^2^ = 0.5137). This correlation increased when the gross lung pathology score of only the five lobes of interest was considered (R^2^ = 0.5903). For the *M. haemolytica*–infected calves, there was also a moderate correlation when the pathology score of the entire lung was considered (R^2^ = 0.4324) and the correlation increased when TUS score was compared to the five lobes of interest (R^2^ = 0.4899). In both infection models, there was a greater correlation between gross lung pathology score and ultrasound score than between gross lung pathology score and clinical score. Thus, TUS provides a more accurate representation of gross lung pathology than clinical scoring and can be obtained at any point in the disease process.

**Figure 9:**
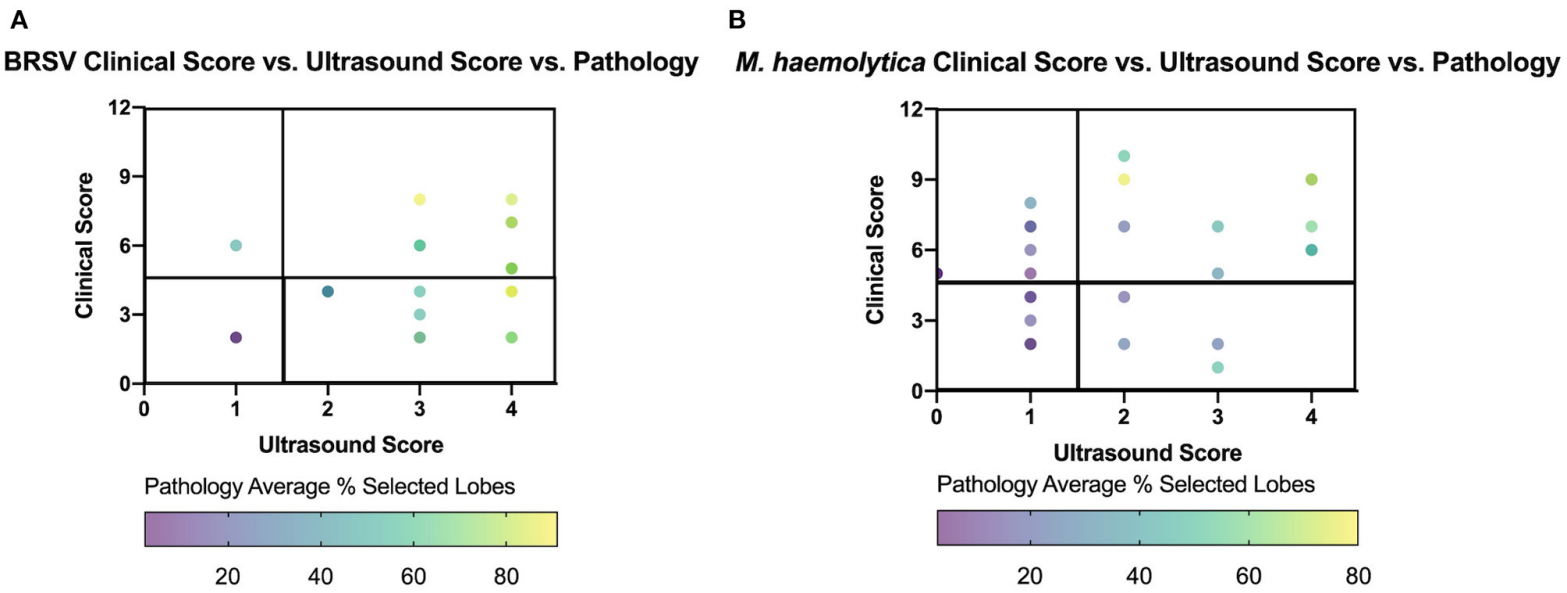
Gross lung pathology plotted against clinical score and ultrasound score on day of necropsy for BRSV-infected **(A)** and *M. haemolytica*–infected calves **(B)**. Clinical score for each calf was plotted against the final ultrasound score with the color of dot representing the percentage of lung affected with gross lesions. The plot is divided into four quadrants, similar to Figure 5.

To further investigate the relationship between clinical score, TUS score, and gross lung pathology score, the three variables were compared in Figure 9. The location of each point on the graph is based on the clinical score and ultrasound score on the day of necropsy, and the focused gross lung pathology score (denoted in Figure 8C) is represented by the color of each data point. For the BRSV-infected calves, a higher gross lung pathology score was associated with a higher TUS score, regardless of clinical score. For the *M. haemolytica*–infected calves, this trend was still present but less pronounced. The differences seen between these two models may be associated with the types of lesions associated with the disease. In BRSV infection, more diffuse and regionalized lesions were observed within the tissue after necropsy. This leads to a higher likelihood of identifying lesions on ultrasound of only a select number of locations. In *M. haemolytica* infection, more localized lesions were observed on necropsy, leading to a decreased likelihood of identifying lesions on ultrasound when only observing select locations. However, there is a trend for both viral and bacterial infections, where, as both clinical and TUS scores increase, the percentage of lung affected increases as well.

## Discussion

There have been many studies on the detrimental effects, both in short and long terms, and the costs of BRD (1, 2, 4). Early detection remains a key factor in mitigating the negative effects of the disease. Previous studies on BRD detection have focused on clinical scoring models that rely on visual indicators, such as nasal discharge, cough, attitude, and physical assessment such as rectal temperature or expiration rate (5). Some of these markers, such as attitude and rectal temperature, are not BRD specific and can be caused by a wide variety of illnesses. In addition, not all calves with BRD will present with the same symptoms or with symptoms that are severe enough to be considered clinically ill. Thus, calves with active, but subclinical, BRD are not identified. In recent studies comparing on-farm detection of clinical disease, TUS has been shown to have increased sensitivity and specificity over clinical scoring, allowing it to capture even subclinical pneumonia (9, 16). Therefore, the goal of our study was to determine whether TUS could provide the same improvements to disease monitoring during experimental BRD trials.

In experimental models of BRD, lung pathology is typically utilized as the gold standard for detection of disease (17–19). For our studies, we compared TUS to lung pathology as the gold standard, and our results show an improved relationship between TUS and lung pathology compared to traditional clinical disease scoring. Thus, TUS was shown to be a more accurate predictor of disease presence within the lungs than clinical scoring. Although the R^2^ value between TUS score and gross lung pathology was only moderate, the TUS data in our study were collected from a small portion of the lung, and using TUS, lesions that are deep to aerated lung cannot be observed. When considering these limitations, the increased correlation between TUS and lung pathology as compared to clinical scoring is increasingly significant. Furthermore, although lung pathology is considered the gold standard, it is highly invasive and requires the animal to be euthanized. In contrast, TUS is non-invasive and can provide results that are well correlated with gross lung pathology, especially if more of the lung field is included in the scan (20), thus providing researchers with an alternative to euthanasia.

The two experimental infection models used in the present study were selected to mimic a viral infection and a bacterial infection due to the known variations in disease presentation and progression in these two types of infection. In most of the parameters, we examined that there was a decreased correlation seen in the *M. haemolytica–*infected calves compared with that in the BRSV-infected calves. This decreased correlation may be related to the increased variation of the disease process itself. Progression of disease in *M. haemolytica* infection models is frequently inconsistent due to variations in endotoxin and leukotoxin production between strains and challenge dose. Previous studies have used doses ranging from 10^6^ to 10^9^ CFU/ml in their inoculation and experienced a range of disease severity even with the same concentration of bacteria (7, 17, 21). Generally, BRSV has a much more consistent pattern of disease (22–24). Within the present study, there was an increased variation in clinical score and TUS score in *M. haemolytica*–infected calves compared with that in BRSV-infected calves. During the Fall 2019 cohort, there was 50% mortality by 48 h after infection, whereas in the Summer 2020 cohort, all infected calves survived to necropsy. When considered together the high variation in clinical score, TUS score and mortality confirm previous observations of the increased variability in disease progression seen in *M. haemolytica* infection. Despite this variation, it is important to note the stronger correlation between TUS score and gross lung pathology compared with the correlation between clinical score and gross lung pathology in *M. haemolytica*–infected calves. Thus, although there was higher variation and somewhat decreased relationships, TUS was still able to accurately capture the differences in disease progression and severity.

The two infection models vary in many ways, but, in addition to the disease mechanisms, there was also variation in the calf populations. In the BRSV infection trial, consolidation was observed before experimental infection in seven of the 32 calves that were entered in the trial. This suggests that these calves had previous respiratory infections that may not have been fully resolved at the time of enrollment in the study. TUS was able to capture the disease in these calves that would have otherwise been missed by clinical scoring alone. The presence of pre-inoculation lesions has the potential to influence all subsequent data that were collected on these calves, in particular TUS and necropsy results, because lesions had already developed before study initiation. Previous studies have also noted suspected pre-enrollment respiratory illness and its effect on the subsequent data that were collected (18, 25). In our study, calves that entered the study with consolidation already present were more likely to remain in the clinical and subclinical pneumonia quadrants. BRD trials with experimental infections are attempting to simplify a complicated disease to study a more specific part of the infection process. Calves that enter experimental trials with undiagnosed or unresolved subclinical respiratory infections can significantly affect study outcomes. Therefore, the results of our study strongly suggest that TUS can be a powerful tool for screening calves for existing disease before they are entered into a trial.

By plotting the relationship between clinical score and TUS score, we identified four different subgroups of calves. Previous papers have identified these calves as having upper respiratory tract infections (positive clinical score and negative TUS score), subclinical pneumonia (negative clinical score and positive TUS score), and clinical pneumonia (positive clinical and TUS score) (8). Because of the nature of our experimental infections, where pathogens were introduced directly into the lungs, the term upper respiratory tract was not used for animals with a positive clinical score that lacked ultrasonographic changes. In our study population, this subset of animals most likely included animals with mild pathology of the lungs or pathology that was either deep to normal aerated lung or in a location that was not visualized using our TUS system. However, we did utilize the “subclinical” and “clinical” terminology for calves that had negative clinical score and positive TUS score (subclinical) and positive clinical and TUS score (clinical). Utilization of these classifications highlights the advantages of using TUS in conjunction with clinical scoring. The calves in the subclinical group represent calves that would have been considered within normal limits if TUS was not used in the study but clearly had changes to their lung tissue that were confirmed on necropsy. It is also important to note that some of the calves within the subclinical pneumonia group may also represent calves who are in the process of disease resolution. Changes in lung tissue that are brought on by pathogens may persist even after the pathogens have been cleared, therefore creating a population of healthy calves with significant abnormalities in lung tissue appearance. The lesions persisting in the lungs of the BRSV-infected calves on day 14 after infection, after the virus has been eliminated from the lungs, are one example of this occurrence. The disease resolution process and its timeline are not well understood, but the presence of long-term effects suggests that the resolution process is likely prolonged compared to the duration of active infection. Disease resolution is an important area for future study of BRD pathogenesis, because it may have long-term implications for animal performance.

The weakest relationship observed was between viral shedding or pathogen burden and TUS score. Our measurements of viral shedding quantified the amount of specific BRSV genes present in nasal secretions on a given day. The peak of BRSV shedding usually occurs around day 6 after infection (23, 26). By day 14 after infection, when the calves in the present study were necropsied, the amount of virus that remains in the tissue is very low or has been completely cleared, as previous studies have shown (23, 26). Therefore, to more accurately reflect pathogen load in the BRSV study, we used the amount of virus shed in nasal secretions on day 7 after infection as our indicator of pathogen burden. Despite selecting the peak shedding day, our dataset still did not detect a correlation with viral shedding. This is not surprising given that viral shedding is not always well correlated with lung damage, which can instead be attributed to the host immune response and subsequent immunopathology (27, 28). In the *M. haemolytica* trials, we used quantitative culture of lung tissue to determine pathogen burden. The presence alone of pathogenic bacteria in the lungs is also not necessarily related to disease severity because some level of *M. haemolytica* presence, which is a normal commensal of the upper respiratory tract, can be considered within normal limits (29, 30). As with viral infection, much of the lung damage resulting from *M. haemolytica* infection can be attributed to immunopathology rather than pathogen burden. Limited work has been done on the relationship between markers of immune function and classifications of BRD as determined by TUS, and all work that has been performed previously has been under field conditions (9, 31).

Most previous research of TUS has focused on its value as a tool to help in clinical diagnosis and treatment of BRD (6, 8, 12). These studies have focused on using TUS in natural BRD infections to confirm its sensitvity and specificity and have primarily taken place in the field where pathogen identification is limited. There are few studies that have used TUS in an experimental infection (7). Our data clearly demonstrate that TUS can provide researchers with additional data on the respiratory health of the animals enrolled in their studies. It is important to note that TUS scanning for clinical diagnosis is typically performed using a different scanning pattern with inclusion of more of the lung surface. In this study, the TUS images were collected by previously untrained individuals; training was able to be completed in a few sessions, and the difficulty of training for image collection was comparable to training for blood collection. It is also important to note that the images were collected as research data to be analyzed and not used to make any clinical diagnosis of disease. When utilized in this manner, TUS can serve as another tool for researchers to use for measuring disease progression, similar to the way that clinical scoring has been integrated in BRD research. In this context, TUS could be especially beneficial in interventional studies that do not call for a final necropsy, such as in the feedlot setting. In these studies, researchers rely on clinical scoring and various blood tests to determine severity of disease (32, 33), and the addition of TUS could provide further information on the health of the lung itself even without necropsy of the animals. Using TUS as a determinant of disease severity could help researchers increase the size of their data pools while decreasing the number of calves that must be euthanized.

## Conclusion

When performed in conjunction with clinical disease scoring, in both controlled infections and field studies, TUS can provide a non-invasive method of assessing disease progression within the lung tissue and provide researchers with a more complete image of calf health. TUS can also identify additional diseased calves that clinical scoring alone cannot capture and provide valuable information on calf lung health at the time of study enrollment.

## Data Availability Statement

The original contributions presented in the study are included in the article/Supplementary Material, further inquiries can be directed to the corresponding author.

## Ethics Statement

The animal study was reviewed and approved by Iowa State University Institutional Care and Use Committee.

## Author Contributions

MP, PM, and JM designed the study. MP and PM collected and analyzed the data. AK provided training and oversight of all TUS-related data collection and interpretation. MP, AK, and JM wrote the manuscript. All authors reviewed the manuscript.

## Funding

This work was funded by NIH grant R21 AI127895 and USDA-NIFA grant 2018-06904 to JM. The funders had no role in study design, data collection and analysis, decision to publish, or preparation of the manuscript.

## Conflict of Interest

The authors declare that the research was conducted in the absence of any commercial or financial relationships that could be construed as a potential conflict of interest.

## Publisher's Note

All claims expressed in this article are solely those of the authors and do not necessarily represent those of their affiliated organizations, or those of the publisher, the editors and the reviewers. Any product that may be evaluated in this article, or claim that may be made by its manufacturer, is not guaranteed or endorsed by the publisher.

## References

[1] USDA. Heifer Calf Health and Management Practices on U.S. Dairy Operations. In: Fort Collins, CO: United States Department of Agriculture, National Veterinary Services Laboratories (2007). 10.3168/jds.2018-15550 31056329

[2] Schneider MJ Tait RG Busby WD Reece JM. An evaluation of bovine respiratory disease complex in feedlot cattle: Impact on performance and carcass traits using treatment records and lung lesion scores. J Anim Sci. (2009) 87:1220–8. 10.2527/jas.2008-1283 19181770

[3] Cramer CM Ollivett TL. Growth of preweaned, group-housed dairy calves diagnosed with respiratory disease using clinical respiratory scoring and thoracic ultrasound-A cohort study. J Dairy Sci. (2019) 102:4322–31. 10.3168/jds.2018-15420 30827549

[4] Stanton AL Kelton DF LeBlanc SJ. Wormuth, J, Leslie KE. The effect of respiratory disease and a preventative antibiotic treatment on growth, survival, age at first calving, and milk production of dairy heifers. J Dairy Sci. (2012) 95:4950–60. 10.3168/jds.2011-5067 22916899

[5] McGuirk SM Peek SF. Timely diagnosis of dairy calf respiratory disease using a standardized scoring system. Anim Health Res Rev. (2014) 15:145–7. 10.1017/S1466252314000267 25410122

[6] Buczinski S Forté G Francoz D Bélanger AM. Comparison of thoracic auscultation, clinical score, and ultrasonography as indicators of bovine respiratory disease in preweaned dairy calves. J Vet Intern Med. (2014) 28:234–42. 10.1111/jvim.12251 24236441 PMC4895545

[7] Baruch J Cernicchiaro N Cull CA Lechtenberg KF Nickell JS Renter DC. Performance of multiple diagnostic methods in assessing the progression of bovine respiratory disease in calves challenged with infectious bovine rhinotracheitis virus and Mannheimia haemolytica. J Anim Sci. (2019) 97:2357–67. 10.1093/jas/skz107 30923802 PMC6541804

[8] Ollivett TL Buczinski S. On-farm use of ultrasonography for bovine respiratory disease. Vet Clin North Am Food Anim Pract. (2016) 32:19–35. 10.1016/j.cvfa.2015.09.001 26922110

[9] Ollivett TL Caswell JL Nydam DV Duffield T Leslie KE Hewson J. Thoracic ultrasonography and bronchoalveolar lavage fluid analysis in holstein calves with subclinical lung lesions. J Vet Intern Med. (2015) 29:1728–34. 10.1111/jvim.13605 26332345 PMC4895683

[10] McGill JL Guerra-Maupome M Schneider S. Prophylactic digoxin treatment reduces IL-17 production in vivo in the neonatal calf and moderates RSV-associated disease. PLoS ONE. (2019) 14:e0214407. 10.1371/journal.pone.0214407 30908540 PMC6433258

[11] Briggs RE Tabatabai LB Tatum FM. Mucosal and parenteral vaccination against pneumonic pasteurellosis in cattle with a modified-live in-frame lktA deletion mutant of Mannheimia haemolytica. Microb Pathog. (2012) 52:302–9. 10.1016/j.micpath.2012.02.008 22401911

[12] Rademacher RD Buczinski S Tripp HM Edmonds MD Johnson EG. Systematic thoracic ultrasonography in acute bovine respiratory disease of feedlot steers: impact of lung consolidation on diagnosis and prognosis in a case-control study. Bov Pract. (2014) 48:1–10. 10.21423/bovine-vol48no1p1-10

[13] McGill JL Kelly SM Kumar P Speckhart S Haughney SL Henningson J. Efficacy of mucosal polyanhydride nanovaccine against respiratory syncytial virus infection in the neonatal calf. Sci Rep. (2018) 8:3021. 10.1038/s41598-018-21292-2 29445124 PMC5813012

[14] Diaz FE Guerra-Maupome M McDonald PO Rivera-Pérez D Kalergis AM McGill JL. Recombinant BCG vaccine is safe and immunogenic in neonatal calves and reduces the clinical disease caused by the respiratory syncytial virus. Front Immunol. (2021) 12:664212. 10.3389/fimmu.2021.664212 33981309 PMC8108697

[15] McGill JL Rusk RA Guerra-Maupome M Briggs RE Sacco RE. Bovine gamma delta T cells contribute to exacerbated IL-17 production in response to co-infection with bovine RSV and Mannheimia haemolytica. PLoS ONE. (2016) 11:e0151083. 10.1371/journal.pone.0151083 26942409 PMC4778910

[16] Berman J Francoz D Dufour S Buczinski S. Bayesian estimation of sensitivity and specificity of systematic thoracic ultrasound exam for diagnosis of bovine respiratory disease in pre-weaned calves. Prev Vet Med. (2019) 162:38–45. 10.1016/j.prevetmed.2018.10.025 30621897

[17] Amrine DE White BJ Larson RL Mosier DA. Pulmonary lesions and clinical disease response to Mannheimia haemolytica challenge 10 days following administration of tildipirosin or tulathromycin. J Anim Sci. (2014) 92:311–9. 10.2527/jas.2013-6577 24243906

[18] Corrigan ME Drouillard JS Spire MF Mosier DA Minton JE Higgins JJ. Effects of melengestrol acetate on the inflammatory response in heifers challenged with Mannheimia haemolytica. J Anim Sci. (2007) 85:1770–9. 10.2527/jas.2006-396 17371793

[19] Amrine DE White BJ Larson R Anderson DE Mosier DA Cernicchiaro N. Precision and accuracy of clinical illness scores, compared with pulmonary consolidation scores, in Holstein calves with experimentally induced Mycoplasma bovis pneumonia. J Am Vet Med Assoc. (2013) 310–5. 10.2460/ajvr.74.2.310 23363359

[20] Pravettoni D Buczinski S Sala G Ferrulli F Bianchi F Boccardo A. Short communication: Diagnostic accuracy of focused lung ultrasonography as a rapid method for the diagnosis of respiratory disease in dairy calves. J Dairy Sci. (2021) 104:4929–35. 10.3168/jds.2020-19377 33663827

[21] Fajt VR Apley MD Roth JA Frank DE Brogden KA Skogerboe TL. The effects of danofloxacin and tilmicosin on neutrophil function and lung consolidation in beef heifer calves with induced Pasteurella (Mannheimia) haemolytica pneumonia. J Vet Pharmacol Ther. (2003) 26:173–9. 10.1046/j.1365-2885.2003.00477.x 12755900

[22] Salt JS Thevasagayam SJ Wiseman A Peters AR. Efficacy of a quadrivalent vaccine against respiratory diseases caused by BHV-1, PI3V, BVDV and BRSV in experimentally infected calves. Vet J. (2007) 174:612–26. 10.1016/j.tvjl.2006.10.007 17276108

[23] Grissett GP White BJ Larson RL. Structured literature review of responses of cattle to viral and bacterial pathogens causing bovine respiratory disease complex. J Vet Intern Med. (2015) 29:770–80. 10.1111/jvim.12597 25929158 PMC4895424

[24] Vangeel I Antonis AF Fluess M Riegler L Peters AR Harmeyer SS. Efficacy of a modified live intranasal bovine respiratory syncytial virus vaccine in 3-week-old calves experimentally challenged with BRSV. Vet J. (2007) 174:627–35. 10.1016/j.tvjl.2006.10.013 17169592

[25] Hanthorn CJ Dewell RD Cooper VL Frana TS Plummer PJ Wang C. Randomized clinical trial to evaluate the pathogenicity of Bibersteinia trehalosi in respiratory disease among calves. BMC Vet Res. (2014) 10:1–8. 10.1186/1746-6148-10-89 24745347 PMC4036748

[26] Bem RA Domachowske JD Rosenberg HF. Animal models of human respiratory syncytial virus disease. Am J Physiol Lung Cell Mol Physiol. (2011) 301:L148–56. 10.1152/ajplung.00065.2011 21571908 PMC3154630

[27] Leite F Kuckleburg C Atapattu D Schultz R Czuprynski CJ. BHV-1 infection and inflammatory cytokines amplify the interaction of Mannheimia haemolytica leukotoxin with bovine peripheral blood mononuclear cells in vitro. Vet Immunol Immunopathol. (2004) 99:193–202. 10.1016/j.vetimm.2004.02.004 15135985

[28] N'jai AU Rivera J Atapattu DN Owusu-Ofori K Czuprynski CJ. Gene expression profiling of bovine bronchial epithelial cells exposed in vitro to bovine herpesvirus 1 and Mannheimia haemolytica. Vet Immunol Immunopathol. (2013) 155:182–9. 10.1016/j.vetimm.2013.06.012 23890750 PMC7127263

[29] Klompmaker AF Brydensholt M Michelsen AM Denwood MJ Kirkeby CT Larsen LE. Estimating clinically relevant cut-off values for a high-throughput quantitative real-time pcr detecting bacterial respiratory pathogens in cattle. Front Vet Sci. (2021) 8:674771. 10.3389/fvets.2021.674771 34113678 PMC8185137

[30] Thomas AC Bailey M Lee MRF Mead A Morales-Aza B Reynolds R. Insights into Pasteurellaceae carriage dynamics in the nasal passages of healthy beef calves. Sci Rep. (2019) 9:1–14. 10.1038/s41598-019-48007-5 31420565 PMC6697682

[31] Cuevas-Gómez I McGee M McCabe M Cormican P O'Riordan E McDaneld T. Growth performance and hematological changes of weaned beef calves diagnosed with respiratory disease using respiratory scoring and thoracic ultrasonography. J Anim Sci. (2020) 98:skaa345 10.1093/jas/skaa345 33095858 PMC7694598

[32] Word AB Broadway PR Burdick Sanchez NS Roberts SL Richeson JT Liang YL. Immune and metabolic responses of beef heifers supplemented with Saccharomyces cerevisiae to a combined viral-bacterial respiratory disease challenge. Transl Anim Sci. (2018) 3:135–48. 10.1093/tas/txy117 32704786 PMC7200475

[33] Kayser WC Carstens GE Washburn KE Welsh TH Lawhon SD Reddy SM. Effects of combined viral-bacterial challenge with or without supplementation of Saccharomyces cerevisiae boulardii strain CNCM I-1079 on immune upregulation and DMI in beef heifers. J Anim Sci. (2019) 97:1171–1. 10.1093/jas/sky483 30597005 PMC6396270

