# Supplementary material for: Use of Thoracic Ultrasonography to Improve Disease Detection in Experimental BRD Infection
Source: Front Vet Sci. 2021 Dec 14;8:763972. doi: 10.3389/fvets.2021.763972 (PMC8712425; doi:10.3389/fvets.2021.763972)
Supplement: Supplementary file 1 [file Data_Sheet_1.docx]

Supplementary Material

# Supplementary Figures

#
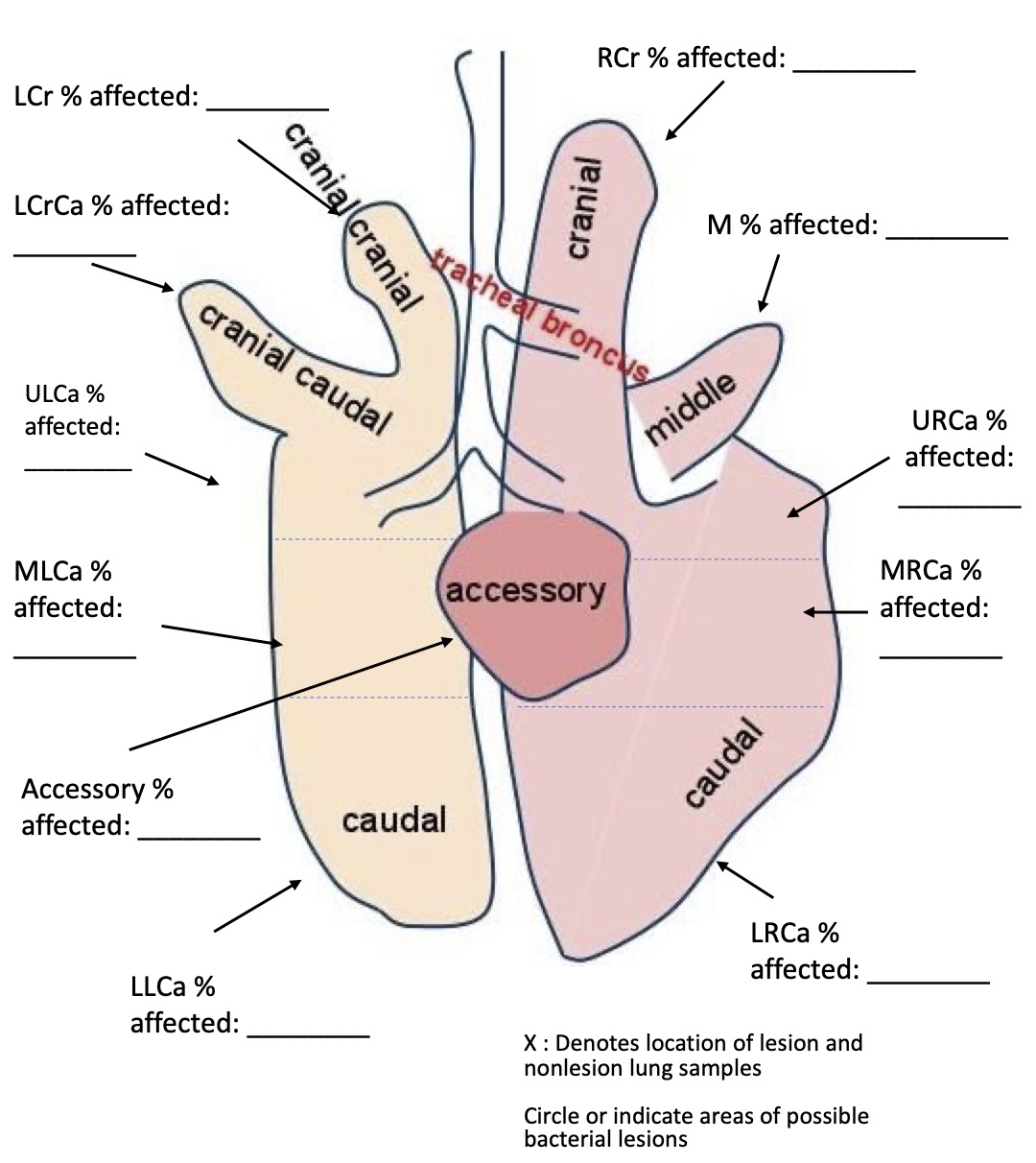


**Supplementary Figure 1.** Gross lung pathology scoring chart. Percent of each lobe affected by lesions is recorded.

**
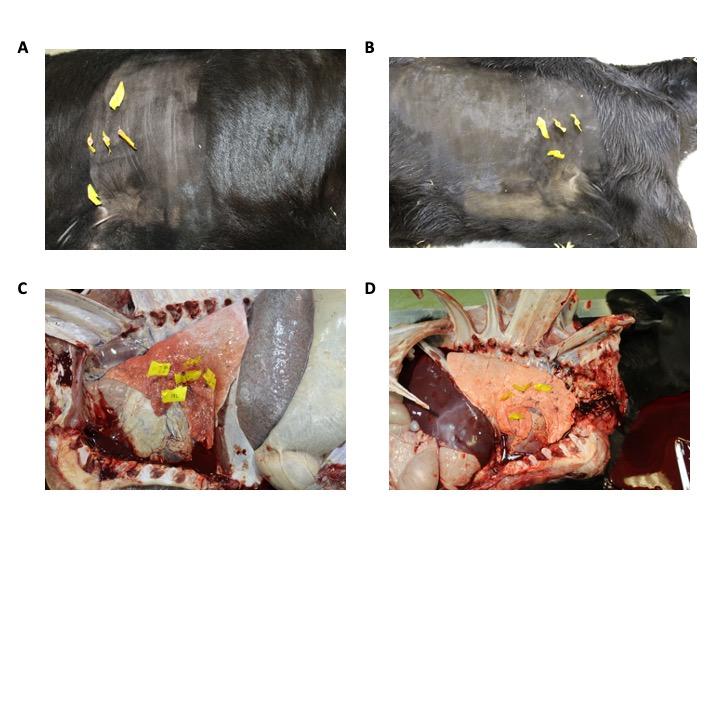
**

**Supplementary Figure 2.** Position of ultrasound locations shown on exterior of calf and lung surface. The yellow pins shown in **(A)** and **(B)** indicate the location of ultrasound images collected on the left and right side of the calf, respectively. Trypan blue dye was injected into the locations of the pin to determine the location of the ultrasound within the lung tissue. **(C)** and **(D)** show the approximate location of the ultrasound image captured on the lung tissue.


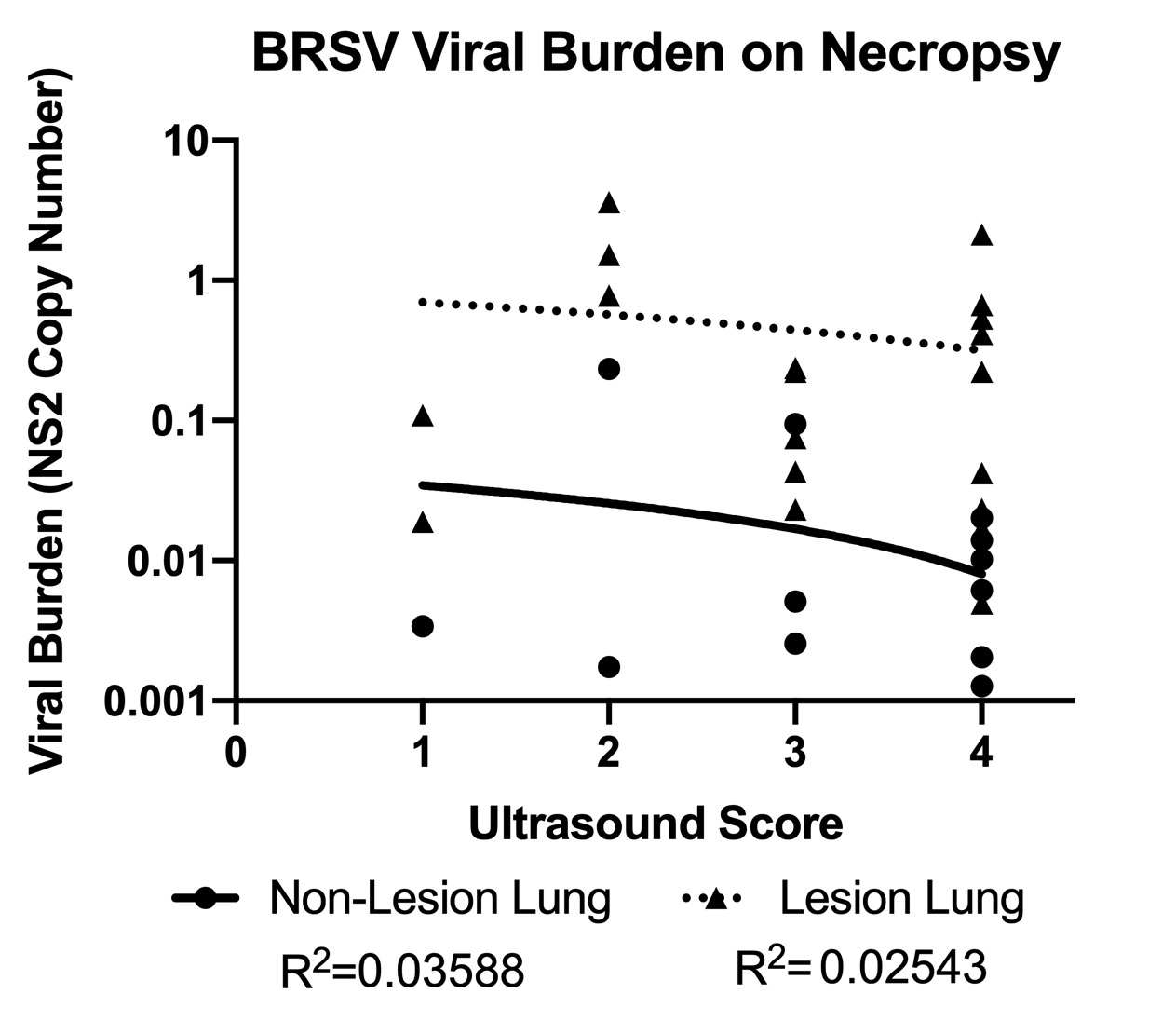


**Supplementary Figure 3.** Relationship between viral load in lung tissue at necropsy and TUS score. qRT-PCR was performed on representative samples of lesioned and non-lesioned lung tissue for the BRSV NS2 gene to determine the viral load present at necropsy.
